# The Prognostic and Predictive Effects of Human Papillomavirus Status in Hypopharyngeal Carcinoma: Population-Based Study

**DOI:** 10.2196/40185

**Published:** 2022-12-16

**Authors:** Shi-Ping Yang, Xiang-Ying Lin, Min Hu, Cheng-Fu Cai

**Affiliations:** 1 Department of Radiation Oncology Hainan General Hospital (Hainan Affiliated Hospital of Hainan Medical University) Haikou China; 2 School of Medicine Xiamen University Xiamen China; 3 Department of Otorhinolaryngology Head and Neck Surgery Zhongshan Hospital, School of Medicine Xiamen University Xiamen China

**Keywords:** hypopharyngeal carcinoma, human papillomavirus, HPV, chemotherapy, radiotherapy, prognosis, cancer, carcinoma

## Abstract

**Background:**

The role of the Human Papillomavirus (HPV) status in patients with hypopharyngeal squamous cell carcinoma (HSCC) remains controversial.

**Objective:**

Our aim was to determine the prognostic and predictive effects of HPV status in patients with locally advanced HSCC (stage III-IVB) receiving primary radiotherapy.

**Methods:**

Patients diagnosed with stage III-IVB HSCC between 2010 and 2016 were identified. HPV status, demographics, clinicopathological characteristics, treatment, and survival data were captured. Kaplan-Meier analysis, multivariable Cox regression analysis, and propensity score matching analysis were performed.

**Results:**

We identified 531 patients in this study and 142 (26.7%) patients with HPV-positive diseases. No significant differences were observed between those with HPV-negative and HPV-positive diseases with regard to demographics, clinicopathological characteristics, and chemotherapy use. HPV-positive HSCC had better head and neck cancer-specific survival (HNCSS; *P*=.001) and overall survival (OS; *P*<.001) compared to those with HPV-negative tumors. Similar results were found using the multivariable Cox regression analysis. Sensitivity analyses showed that the receipt of chemotherapy was associated with significantly improving HNCSS (*P*<.001) and OS (*P*<.001) compared to not receiving chemotherapy in HPV-negative HSCC, whereas comparable HNCSS (*P*=.59) and OS (*P*=.12) were found between both treatment arms in HPV-positive HSCC. Similar results were found after propensity score matching.

**Conclusions:**

Approximately one-quarter of HSCC may be HPV-related, and HPV-positive HSCC is associated with improved survival outcomes. Furthermore, additional chemotherapy appears to be not related to a survival benefit in patients with HPV-positive tumors who received primary radiotherapy.

## Introduction

Head and neck squamous cell carcinoma (HNSCC) accounts for more than 90% of all head and neck malignancies [1]. However, hypopharyngeal squamous cell carcinoma (HSCC) is relatively rare overall, accounting for approximately 3% of all HNSCCs [2]. Symptoms and signs of HSCC tend to be minimized by patients until severe distress occurs or an obvious mass is found in the neck. Because of this, most patients are diagnosed with locally advanced HNSCC (stage III or IV) [3], with a 5-year overall survival (OS) of 30%, which has the worst prognosis compared to other sites of HNSCC [2].

Human papillomavirus (HPV) is an important factor in the carcinogenesis of HNSCC, especially in oropharyngeal squamous cell carcinoma (OSCC) [4]. The existing evidence has shown a better prognosis in patients with HPV-positive OSCC. Moreover, HPV status has also impacted the treatment decision-making of OSCC [5,6]. Several studies with a small sample size suggested that the HPV infection rate in HSCC was relatively low (1.6%-8.5%) [7-9]. However, two recent population-based studies indicated that 17.7%-23.9% of patients with HSCC had HPV-positive diseases [10,11]. This raises our question of whether HPV status has a prognostic effect on HSCC. Currently, contradictory results were observed in several previous studies regarding the prognosis of HPV status in HSCC, with some studies indicating HPV-related HSCC with significantly improved survival outcomes, whereas others have found similar survival rates between HPV-negative and HPV-positive diseases [7,10-15]. In addition, there are limited studies assessing the role of HPV status in patients who received primary radiotherapy or chemoradiotherapy regarding the HSCC [12]. In light of this, we conducted this analysis from the Surveillance, Epidemiology, and End Results (SEER) database to investigate the prognostic and predictive effect of HPV status in stage III-IVB HSCC receiving primary radiotherapy.

## Methods

### Patients Selection Criteria

The data for this study were captured from the SEER database [16], in which HPV status was categorized as either HPV-negative, HPV-positive, or unknown status. HPV status was determined by p16-immunohistochemistry, in situ hybridization, or polymerase chain reaction methods of pathologic specimens from either the primary hypopharyngeal tumors or metastatic cervical lymph nodes. The HPV data set was reviewed by the SEER data quality team to ensure the accuracy of HPV testing status [17].

We queried the SEER public database from 2010 to 2016 for patients diagnosed with stage III-IVB HSCC who received primary radiotherapy with or without chemotherapy. We did not include patients diagnosed before 2010 because HPV status was only added as a SEER variable in 2010. Those with unknown HPV status were excluded. In addition, we excluded patients who were treated with non-beam irradiation, including radioactive implants and radioisotopes.

### Ethical Considerations

This study was approved by the ethics committee of the Hainan General Hospital (No. ZDYF2022SHFZ130).

### Data Collection

The following demographics, clinicopathological characteristics, or treatment data were identified from each patient’s medical record: age, gender, race, grade, tumor location, American Joint Committee on Cancer (AJCC) staging, HPV status, chemotherapy use, insurance status, and marital status. AJCC 7th staging system was used to determine the stage of patients. The primary outcome endpoints were head and neck cancer-specific survival (HNCSS) and OS. HNCSS was defined as the time from the initial diagnosis of HCSS till death due to head and neck cancer. OS was defined as the time from the initial diagnosis of HCSS till death from all causes.

### Statistical Analyses

The difference in patients’ characteristics and treatment data were compared using the chi-square test or Fisher exact test. HNCSS and OS curves were estimated using the Kaplan-Meier methods and compared by the log-rank test. A 1:1 propensity score matching (PSM) was conducted to balance the potential confounders. Multivariable Cox regression models were used to investigate whether HPV-positive HSCC was related to better HNCSS and OS. Sensitivity analyses were used to investigate the effect of chemotherapy on survival according to HPV status. Data analyses were conducted using SPSS statistical software (version 22.0; IBM Corp). *P*<.05 was considered to be statistically significant.

## Results

### Baseline Characteristics

**Figure 1:**
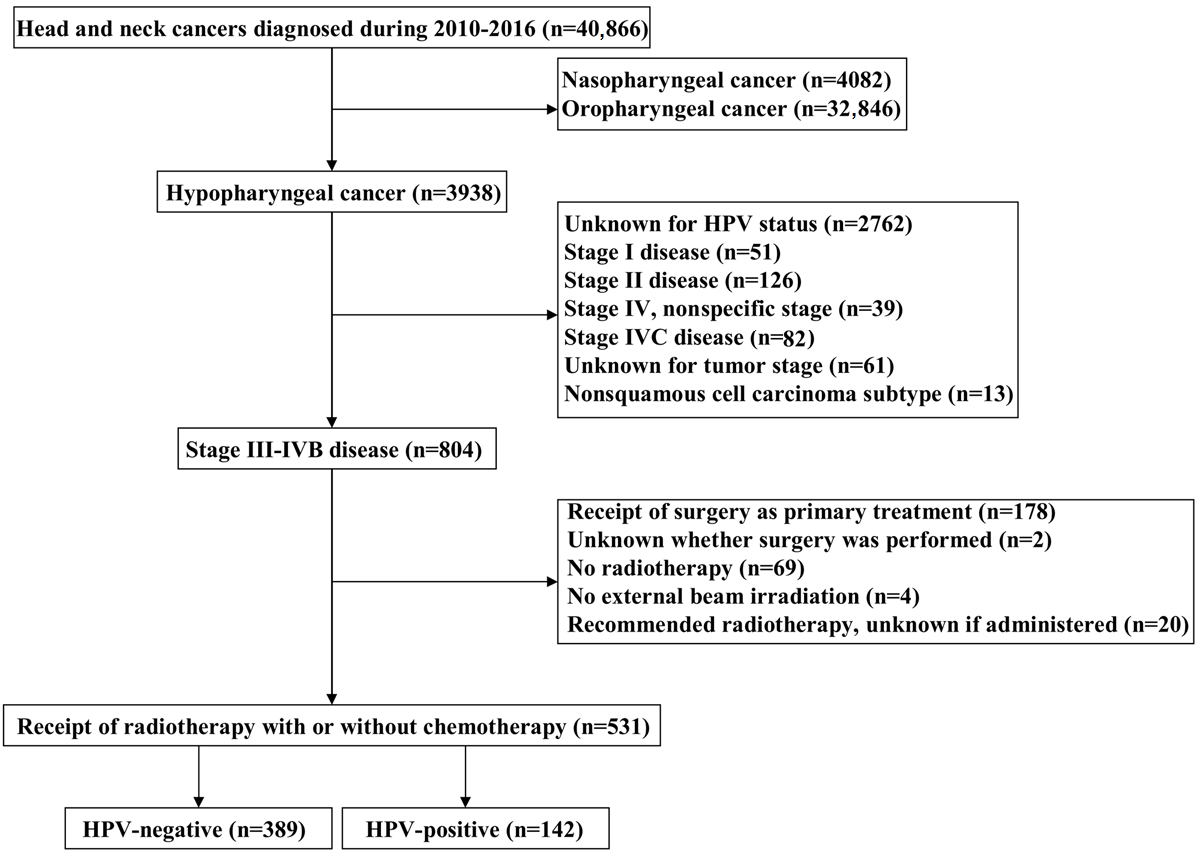
Patient selection procedure in the Surveillance, Epidemiology, and End Results (SEER) database regarding human papillomavirus (HPV) status in head and neck cancers.

We identified 531 patients in this study (Figure 1), including 389 (73.3%) patients with HPV-negative diseases and 142 (26.7%) patients who had HPV-positive HSCC. Table 1 lists the baseline characteristics of the study cohort. A total of 445 (83.8%) patients were male; 404 (76.1%) patients had stage IVA-IVB disease; and 466 (87.8%) patients received chemotherapy. In patients with tumor location available (n=381), 74% (n=282) of the tumor was located in pyriform sinus. No significant difference was found between HPV-negative and HPV-positive diseases with regard to age, gender, race, AJCC staging, tumor grade, tumor location, chemotherapy use, insurance status, and marital status. Patients of younger age (*P*=.002) and Non-Hispanic White patients (*P*=.02) were more likely to receive chemotherapy (Table S1 in Multimedia Appendix 1).

**Table 1:** Patients’ baseline characteristics according to human papillomavirus (HPV) status.

Variables		Patients, n	HPV-negative (n=389), n (%)	HPV-positive (n=142), n (%)	*P* value
**Age (years), n (%)**					.73
	<50	40	28 (7.2)	12 (8.5)	
	50-64	242	181 (46.5)	61 (43)	
	>64	249	180 (46.3)	69 (48.6)	
**Gender, n (%)**					.79
	Male	445	325 (83.5)	120 (84.5)	
	Female	86	64 (16.5)	22 (15.5)	
**Race, n (%)**					.06
	Non-Hispanic White	379	267 (68.6)	112 (78.9)	
	Non-Hispanic Black	65	54 (13.9)	11 (7.7)	
	Hispanic (all)	47	34 (8.7)	13 (9.2)	
	Other	40	34 (8.7)	6 (4.2)	
**Grade, n (%)**					.19
	Well differentiated	20	17 (5.7)	3 (2.9)	
	Moderately differentiated	201	155 (52)	46 (45.1)	
	Poorly differentiated or undifferentiated	179	126 (42.3)	53 (52)	
	Unknown	131	—^a^	—	
**Tumor location, n (%)**					.18
	Pyriform sinus	282	205 (71.7)	77 (81.1)	
	Aryepiglottic fold	40	30 (10.5)	10 (10.5)	
	Postcricoid region	12	11 (3.8)	1 (1.1)	
	Posterior wall	47	40 (14)	7 (7.4)	
	Unknown	150	—	—	
**AJCC^b^ stage, n (%)**					.85
	III	127	95 (24.4)	32 (22.5)	
	IVA	335	245 (63)	90 (63.4)	
	IVB	69	49 (12.6)	20 (14.1)	
**Chemotherapy, n (%)**					.91
	No	65	48 (12.3)	17 (12)	
	Yes	466	341 (87.7)	125 (88)	
**Insurance status, n (%)**					.05
	Insured	22	20 (5.2)	2 (1.4)	
	Uninsured	503	363 (94.8)	140 (98.6)	
	Unknown	6	—	—	
**Marital status, n (%)**					.32
	Married	253	180 (48.8)	73 (54.1)	
	Divorced	86	68 (18.4)	18 (13.3)	
	Single	128	91 (24.7)	37 (27.4)	
	Widowed	37	30 (8.1)	7 (5.2)	
	Unknown	27	—	—	

^a^Not available.

^b^AJCC: American Joint Committee on Cancer.

### Survival Outcomes and Prognostic Analyses Stratified by HPV Status

**Figure 2:**
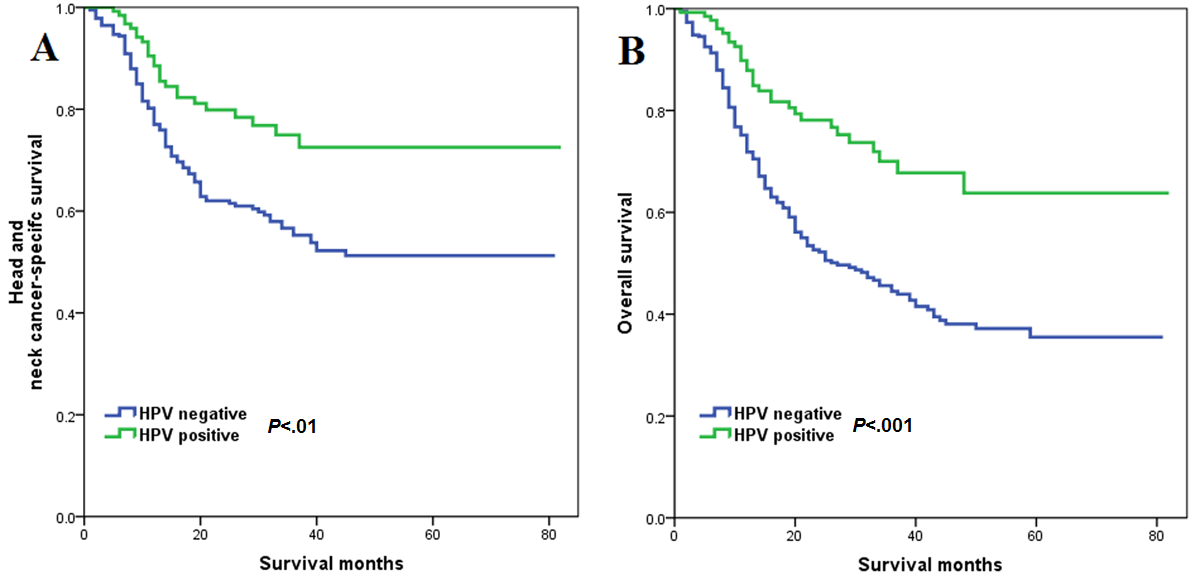
Survival curve in hypopharyngeal squamous cell carcinoma according to human papillomavirus (HPV) status.

With a median follow-up of 16 (range 0-82) months, a total of 205 deaths were observed, including 152 patients who died of head and neck cancer-related disease. Using Kaplan-Meier survival estimates, HPV-positive patients had better survival outcomes compared to HPV-negative patients. The 3-year HNCSS in HPV-negative and HPV-positive patients was 55.3% and 74.9% (*P=*.001), respectively (Figure 2A). The 3-year OS in HPV-negative and HPV-positive patients was 44.5% and 70% (*P<*.001), respectively (Figure 2B).

Table 2 lists the results of multivariate Cox regression analyses. The results indicated that patients with positive HPV had significantly better HNCSS (hazard ratio [HR]: 0.460; *P*<.001) and OS (HR: 0.422; *P*<.001) compared to patients with negative HPV. In addition, patients who received chemotherapy had better HNCSS (HR: 0.405; *P*<.001) and OS (HR: 0.405; *P*<.001) than those without chemotherapy. Moreover, age, race, tumor location, AJCC stage, and marital status were also risk factors independently associated with HNCSS or OS.

**Table 2:** Multivariable Cox regression analyses for factors affecting survival outcomes.

Variables		head and neck cancer-specific survival			overall survival		
		HR^a^	95% CI	*P* value	HR	95% CI	*P* value
**Age** **(years)**							
	<50	1	—^b^	—	1	—	—
	50-64	1.528	0.751-3.110	.24	1.363	0.752-2.468	.31
	>64	2.240	1.104-4.547	.03	2.025	1.119-3.664	.02
**Gender**							
	Male	1	—	—	1	—	—
	Female	1.128	0.720-1.768	.60	1.118	0.759-1.647	.57
**Race**							
	Non-Hispanic White	1	—	—	1	—	—
	Non-Hispanic Black	1.419	0.869-2.317	.16	1.841	1.253-2.704	.002
	Hispanic (all)	1.116	0.618-2.017	.72	1.034	0.620-1.725	.90
	Other	1.374	0.776-2.433	.28	1.203	0.725-1.999	.48
**Grade**							
	Well differentiated	1	—	—	1	—	—
	Moderately differentiated	0.712	0.338-1.503	.37	0.604	0.316-1.152	.13
	Poorly differentiated or undifferentiated	0.684	0.324-1.448	.32	0.613	0.321-1.170	.14
**Tumor location**							
	Pyriform sinus	1	—	—	1	—	—
	Aryepiglottic fold	1.484	0.789-2.790	.22	1.355	0.782-2.347	.28
	Postcricoid region	2.413	1.028-5.662	.04	1.746	0.769-3.967	.18
	Posterior wall	1.366	0.797-2.340	.26	1.291	0.811-2.056	.28
**AJCC^c^ stage**							
	III	1	—	—	1	—	—
	IVA	1.676	1.085-2.587	.02	1.445	1.011-2.065	.04
	IVB	2.513	1.408-4.484	.002	2.252	1.382-3.669	.001
**Chemotherapy**							
	No	1	—	—	1	—	—
	Yes	0.405	0.259-0.633	<.001	0.405	0.274-0.598	<.001
**Insurance status**							
	Insured	1	—	—	1	—	—
	Uninsured	0.721	0.355-1.464	.37	0.788	0.416-1.489	.46
**Marital status**							
	Married	1	—	—	1	—	—
	Divorced	1.016	0.621-1.663	.95	1.087	0.724-1.631	.69
	Single	1.728	1.143-2.612	.009	1.323	0.904-1.937	.15
	Widowed	1.658	0.910-3.020	.10	1.719	1.032-2.864	.04
**HPV^d^ status**							
	HPV-negative	1	—	—	1	—	—
	HPV-positive	0.460	0.299-0.709	<.001	0.422	0.285-0.625	<.001

^a^HR: hazard ratio.

^b^Not applicable.

^c^AJCC: American Joint Committee on Cancer.

^d^HPV: human papillomavirus.

### Effect of Chemotherapy After Stratification of HPV Status

**Figure 3:**
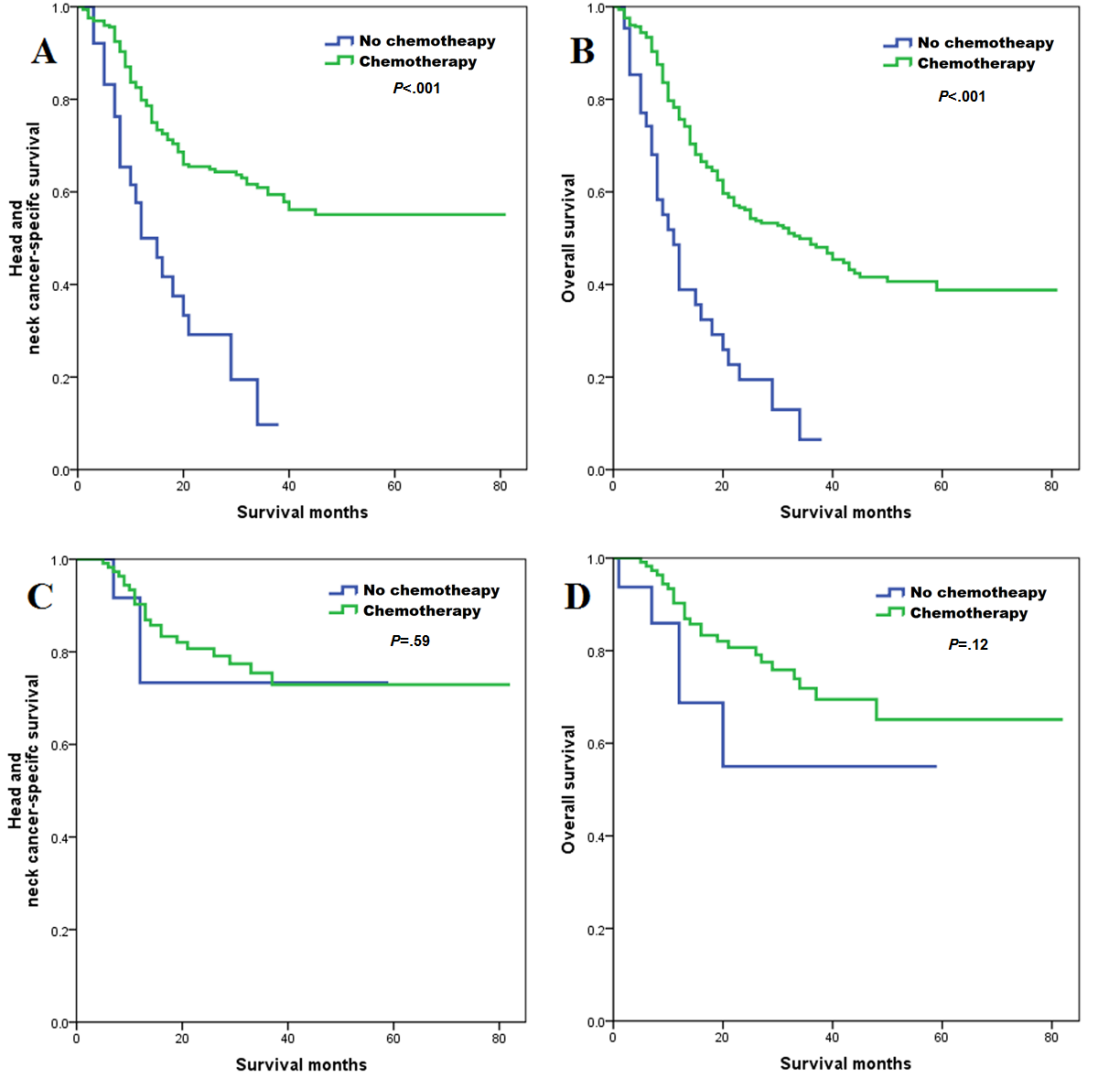
The effect of additional chemotherapy—according to human papillomavirus (HPV) status—before propensity score matching on head and neck cancer-specific survival (A: HPV-negative; C: HPV-positive) and overall survival (B: HPV-negative; D: HPV-positive) among patients with hypopharyngeal squamous cell carcinoma.

**Figure 4:**
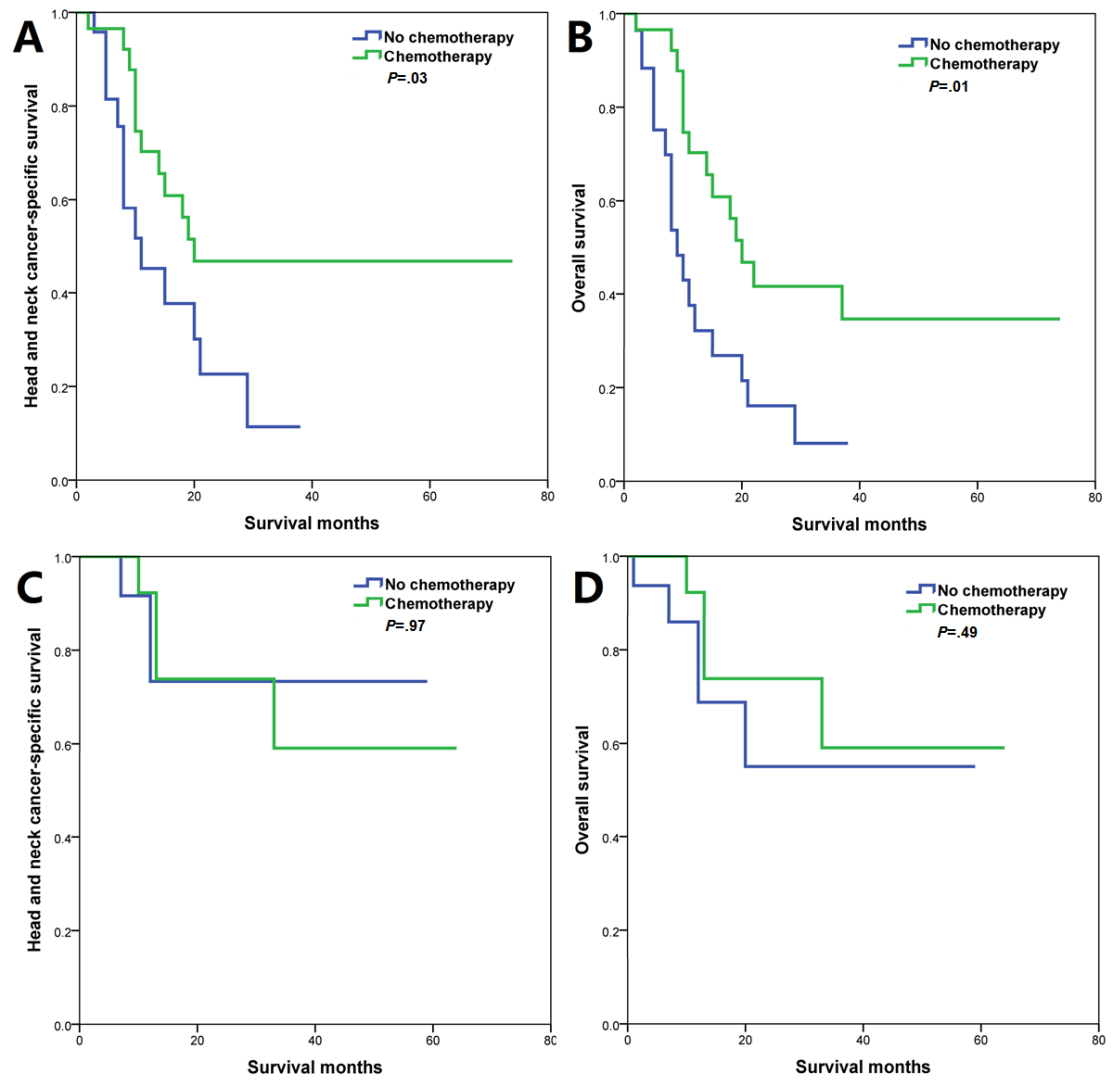
The effect of additional chemotherapy—according to human papillomavirus (HPV) status—after propensity score matching on head and neck cancer-specific survival (A: HPV-negative; C: HPV-positive) and overall survival (B: HPV-negative; D: HPV-positive) among patients with hypopharyngeal squamous cell carcinoma.

Sensitivity analyses were used to investigate the effect of chemotherapy according to HPV status. After adjustment of age, gender, race, grade, tumor location, AJCC stage, insurance status, and marital status, the results of multivariate Cox regression analyses indicated that receipt of chemotherapy was related to better HNCSS (HR: 0.350; *P*<.001) and OS (HR: 0.342; *P*<.001) compared to not receiving chemotherapy in patients with HPV-negative HSCC, while similar HNCSS (chemotherapy vs no chemotherapy: HR 0.581; *P*=.45) and OS (chemotherapy vs no chemotherapy: HR 0.340; *P*=.07) were observed between both treatment arms in HPV-positive HSCC (Table 3). There were 31 (Table S2 in Multimedia Appendix 1) and 17 (Table S3 in Multimedia Appendix 1) pairs of patients matched using PSM in HPV-negative and HPV-positive groups, respectively. The survival curves by chemotherapy receipt according to HPV status are listed in Figure 3. Similar results were found after PSM (Table 3 and Figure 4).

**Table 3:** Multivariable Cox regression analyses to determine the effect of chemotherapy on survival outcomes according to HPV status.

Variables			Head and neck cancer-specific survival			Overall survival		
			HR^a^	95% CI	*P* value	HR	95% CI	*P* value
**HPV^b^- negative (before PSM^c^)**								
	**Chemotherapy**							
		No	1	—^d^	—	1	—	—
		Yes	0.350	0.216-0.567	<.001	0.342	0.224-0.523	<.001
**HPV** **-** **positive** **(before PSM)**								
	**Chemotherapy**							
		No	1	—	—	1	—	—
		Yes	0.581	0.143-2.354	.45	0.340	0.106-1.087	.07
**HPV** **-negative (after PSM)**								
	**Chemotherapy**							
		No	1	—	—	1	—	—
		Yes	0.445	0.204-0.971	.04	0.395	0.194-0.803	.01
**HPV** **-** **positive** **(after PSM)**								
	**Chemotherapy**							
		No	1	—	—	—	—	—
		Yes	0.048	0.002-1.307	.07	—	—	.29

^a^HR: hazard ratio.

^b^HPV: human papillomavirus.

^c^PSM: propensity score matching.

^d^Not applicable.

## Discussion

### Principal Findings

In this study, we first investigated the prognostic and predictive effects of HPV status of locally advanced HSCC receiving definitive radiotherapy. Our results showed that 26.7% of patients with locally advanced HSCC had HPV-positive disease. Moreover, patients with positive HPV had a better prognosis than those with negative HPV. The secondary objective of this study was to investigate whether the HPV status could predict the effect of chemotherapy on survival in patients with HSCC receiving radiotherapy. The sensitivity analyses showed that the addition of chemotherapy only improved survival outcomes in HPV-negative HSCC, but not in HPV-positive HSCC.

The etiological relation with cancers developing in the nonoropharynx parts versus the oropharynx remains unestablished. The incidence of HPV infection created a significant difference regarding tumor sites and race. In a large cohort study [10] from the National Cancer Data Base including 24740 patients with HNSCC, the percentages of HPV-positive disease by tumor location were 17.7% for hypopharynx, 11% for larynx, 10.6% for oral cavity, and 62.9% for oropharynx. A study from Japan [18] included 493 patients with HNSCC, in whom the prevalence of HPV in oropharyngeal, oral, nasopharyngeal, hypopharyngeal, and laryngeal carcinomas was 34.4%, 0%, 12%, 3.5%, and 3.9%, respectively. Another study from Thailand [8] showed that the prevalence of HPV in OSCC was only 6%, and no HPV infection was found in laryngeal and hypopharyngeal cancers. However, a case-control study in the Southern Chinese population showed that 29.4% of oropharyngeal cancers, 16.1% of laryngeal cancers, 14.3% of hypopharyngeal cancers, and 2.2% of oral cavity cancers were HPV DNA positive [19]. In our study, the incidence of HPV-related disease in HSCC was higher than that in the above studies (26.7% vs 3.3%-17.7%), which might be due to the fact that we only included patients in stage III-IVB receiving primary radiotherapy or chemoradiotherapy. Similar results were found in OSCC, which also showed a higher percentage of HPV-related patients receiving primary radiotherapy or chemoradiotherapy [20]. In addition, the patient selection, the geographical distribution of patients, and the HPV testing methods also played a role in this variability. Moreover, the probability of tobacco use in different cohorts may also lead to a discrepancy in HPV infection rates [9].

Patients with HPV-positive OSCC were more likely to be male, younger, in an early tumor stage, and in advanced nodal stage [21]. In a recent SEER study including stage I-IV HSCC, they found a higher proportion of HPV-positive patients who were White or Hispanic [22]. Another SEER study by Abdel-Rahman [11] indicated that HPV-related HSCC was more likely to involve younger people and higher tumor grades. The Danish Head and Neck Cancer Group trials also found no significant difference regarding age and AJCC stage in laryngeal and hypopharyngeal cancers between p16-negative and p16-positive disease, while the p16-positive disease was more likely to present in female patients (29% vs 17%; *P*=.02) [13]. In our study, we also showed that a higher proportion of HPV-related tumors were found in Non-Hispanic White patients; otherwise, no significant differences were found in the demographics, clinicopathological characteristics, or chemotherapy receipt between HPV-negative and HPV-positive diseases.

The role of the HPV status in HSCC remains controversial. Hughes et al [12] included 94 patients with laryngeal or hypopharyngeal cancers (13% of patients were HPV-related), and HPV did not appear to significantly impact survival or disease control in patients in stage III-IV receiving primary chemoradiotherapy. In addition, a study from Karolinska Institute [7] included 82 patients with HSCC and found that being HPV DNA positive (n=7) was associated with better OS but not disease-specific survival compared to those being HPV DNA negative, while a similar prognosis was found between p16-negative and p16-positive diseases. Several studies including the Danish Head and Neck Cancer Group trials also showed similar outcomes between p16-negative and p16-positive diseases, suggesting that the prognostic effect may be limited to OSCC only [13-15]. However, a small portion of patients with HPV DNA-positive or p16-positive disease in the above studies limited the study to be applied to the general population. Two larger cohort studies from the National Cancer Data Base (n=1085) and SEER (n=1157) included patients with HSCC, and they found that those with HPV-positive HSCC had better OS and cancer-specific survival compared to those with HPV-negative HSCC [10,11]. To our knowledge, our study was the largest cohort study to investigate the role of HPV status in patients with HSCC receiving primary radiotherapy or chemoradiotherapy. Within this cohort, we suggest that HPV status may be an additional factor for risk stratification of HSCC, and the future revision of the AJCC staging should consider the HPV status.

HPV-positive OSCC is a distinct pathological entity and may deserve a more personalized therapeutic strategy to decrease the severe early and late toxicities, including de-escalation of radiation doses, less toxic chemotherapy treatment, and removal of chemotherapy [23-25]. In HPV-positive HNSCC, the data also suggested that intensive chemoradiotherapy approaches did not improve clinical outcomes compared to radiotherapy alone in the definitive radiotherapy setting and postoperative radiotherapy setting [26-29]. In National Comprehensive Cancer Network Clinical Practice Guidelines in Oncology, the optimal treatment options for HSCC are induction chemotherapy, surgery, concurrent chemoradiotherapy, or clinical trials [30]. Radiotherapy or chemoradiotherapy is not recommended as primary treatment. However, in clinical practice, concurrent chemoradiotherapy remains the main treatment strategy for organ preservation among patients with HSCC. Two previous studies including patients from the SEER and National Cancer Database showed that 81.3% and 72.2% of patients have received radiotherapy or chemoradiotherapy, respectively [31,32]. In this study, we demonstrated a survival benefit of additional chemotherapy in HPV-negative HSCC, whereas radiotherapy outcome did not differ by the receipt of chemotherapy in HPV-positive HSCC. One possible explanation for this finding is that HPV-positive HSCC may be cured by primary radiotherapy. The results of HPV status have been widely used for prognostic assessment and treatment decision-making for OSCC. Our results indicated that the prognosis and treatment response for HSCC could also be individualized according to HPV status.

The reasons for the better prognosis and limited effect from chemotherapy in patients with HPV-related HSCC who received primary radiotherapy remain unsolved. The high response of HPV-associated cancer cells to radiotherapy may be related to cell cycle dysregulation, repopulation signaling, and impaired DNA repair capacity of the tumor cells [33-36]. Furthermore, the proximity of the HNSCC to lymphoid tissues may also contribute to the high radiosensitivity of HPV-related tumors, and the interaction between the virus antigens and the immune system may contribute to enhancing the radiosensitivity [13]. Whether HPV-related HSCC also possesses such enhanced radiosensitivity remains to be clarified. In addition, HPV-negative tumors often carry frequent TP53 mutations, resulting in significant radioresistance [37]. Moreover, those with HPV-positive HSCC had a better prognosis due to higher immune activity and overexpression of immune proteins compared to those with HPV-negative HSCC, which was similar to the results for OSCC [38-40].

Several limitations should be acknowledged in this study. First, the findings of our study should be viewed with caution because this is a retrospective observational study from a population-based cohort. Second, the rationale for treatment decisions among each patient group cannot be ascertained from the SEER database. Third, the HPV testing results may be heterogeneous with respect to technique, and the results of HPV testing were not centrally reviewed. However, a previous study showed high concordance among the p16-immunohistochemistry, in situ hybridization, or polymerase chain reaction methods for detecting HPV status [41]. Fourth, several confounding factors were not measured in the SEER database, including the chemotherapy regimen, the target volume of radiotherapy, the sequence of chemotherapy and radiotherapy, details regarding patient performance status, and tobacco or alcohol exposure. Moreover, the patterns of locoregional and distant metastasis after primary radiotherapy or chemoradiotherapy were also not routinely captured in the SEER database. Finally, approximately 15% of patients should have undergone salvage surgery for recurrence after radiotherapy or chemoradiotherapy in a previous study [42]. However, SEER does not record the information regarding salvage surgery, thus it is unable to evaluate the impact of salvage surgery on the prognosis and its distribution in both groups (HPV-positive and HPV-negative). Despite the above limitations, we believe the results from our population-based study are provocative enough to warrant further investigation of the prognostic and predictive effects of the HPV status in HSCC.

### Conclusions

In conclusion, our study suggests that approximately one-quarter of HSCC may be HPV-related, and HPV-positive HSCC is associated with improved survival outcomes. Furthermore, additional chemotherapy appears to be not related to a survival benefit in HPV-positive tumors receiving primary radiotherapy. More studies are required to better understand the prognostic and predictive effects of the HPV status in HSCC.

## Appendix

Multimedia Appendix 1 Supplementary tables.

## Abbreviations

AJCC: American Joint Committee on Cancer
HNCSS: head and neck cancer-specific survival
HNSCC: head and neck squamous cell carcinoma
HPV: human papillomavirus
HR: hazard ratio
HSCC: hypopharyngeal squamous cell carcinoma
OS: overall survival
OSCC: oropharyngeal squamous cell carcinoma
PSM: propensity score matching
SEER: Surveillance, Epidemiology, and End Results
