# Supplementary material for: The Prognostic and Predictive Effects of Human Papillomavirus Status in Hypopharyngeal Carcinoma: Population-Based Study
Source: JMIR Public Health Surveill. 2022 Dec 16;8(12):e40185. doi: 10.2196/40185 (PMC9804097; doi:10.2196/40185)
Supplement: Multimedia Appendix 1 [file publichealth_v8i12e40185_app1.pdf]

Table S1. Patient baseline characteristics between those with and without chemotherapy.

Variables	n	No chemotherapy	Chemotherapy	*P*
Age (years)				
<65	282	23(35.4)	259(55.6)	<.01
≥65	249	42(64.6)	207(44.4)	
Gender				
Male	445	53(81.5)	392(84.1)	.59
Female	86	12(18.5)	74(15.9)	
Race/Ethnicity				
Non-Hispanic White	379	44(67.7)	335(71.9)	.02
Non-Hispanic Black	65	15(23.1)	50(10.7)	
Hispanic (all)	47	3(4.6)	44(9.4)	
Other	40	3(4.6)	37(7.9)	
Grade				
Well differentiated	20	3(4.6)	17(3.6)	.06
Moderately differentiated	201	29(44.6)	172(36.9)	
Poorly/undifferentiated	179	19(29.2)	160(34.3)	
Unknown	31	14(21.5)	117(25.1)	
Tumor location				
Pyriform sinus	282	37(56.9)	245(52.6)	.75
Aryepiglottic fold	40	3(4.6)	37(7.9)	
Postcricoid region	12	2(3.1)	10(2.1)	
Posterior wall	47	7(10.8)	40(8.6)	
Unknown	150	16(24.6)	134(28.8)	
AJCC stage				
III	127	22(33.8)	105(22.5)	.05
IVA	335	39(60.0)	296(63.5)	
IVB	69	4(6.2)	65(13.9)	
Insurance status				
Insured	503	63(96.9)	440(94.4)	.51
Uninsured	22	1(1.5)	21(4.5)	
Unknown	6	1(1.5)	5(1.1)	
Marital status				
Married	253	23(35.4)	230(49.4)	.09
No married *	252	37(56.9)	215(46.1)	
Unknown	26	5(7.7)	21(4.5)	
HPV status				
HPV negative	389	48(26.2)	341(26.8)	.91
HPV positive	142	17(73.8)	125(73.2)	

AJCC, American Joint Committee on Cancer; HPV, human papillomavirus.
*indicates those divorced, single, or widowed.

Table S2. Patient baseline characteristics between those with and without chemotherapy in human papillomavirus negative patients after propensity score matching.

Variables	n	No chemotherapy	Chemotherapy	*P*
Age (years)				
<65	23	12	11	.73
≥65	39	9	20	
Gender				
Male	54	28	26	.49
Female	8	3	5	
Race/Ethnicity				
Non-Hispanic White	41	20	21	.71
Non-Hispanic Black	13	8	5	
Hispanic (all)	2	1	1	
Other	6	2	4	
Grade				
Well differentiated	1	1	0	.61
Moderately differentiated	27	15	12	
Poorly/undifferentiated	21	9	12	
Unknown	13	6	7	
Tumor location				
Pyriform sinus	39	20	19	.51
Aryepiglottic fold	2	0	2	
Postcricoid region	1	0	1	
Posterior wall	6	3	3	
Unknown	14	8	6	
AJCC stage				
III	16	9	7	.79
IVA	41	20	21	
IVB	5	2	3	
Insurance status				
Insured	60	30	30	.37
Uninsured	1	1	0	
Unknown	1	0	1	
Marital status				
Married	35	19	16	.49
No married*	26	12	14	
Unknown	0	0	0	

AJCC, American Joint Committee on Cancer.

* indicates those divorced, single, or widowed.

Table S3. Patient baseline characteristics between those with and without chemotherapy in human papillomavirus positive patients after propensity score matching.

Variables	n	No chemotherapy	Chemotherapy	*P*
Age (years)				
<65	16	6	10	.17
≥65	18	11	7	
Gender				
Male	28	15	13	.37
Female	6	2	4	
Race/Ethnicity				
Non-Hispanic White	23	14	9	.29
Non-Hispanic Black	5	2	3	
Hispanic (all)	4	1	3	
Other	2	0	2	
Grade				
Well differentiated	1	0	1	.47
Moderately differentiated	18	9	9	
Poorly/undifferentiated	8	3	5	
Unknown	7	5	2	
Tumor location				
Pyriform sinus	16	9	7	.69
Aryepiglottic fold	3	1	2	
Postcricoid region	1	1	0	
Posterior wall	14	6	8	
Unknown				
AJCC stage				
III	8	6	2	.13
IVA	24	11	13	
IVB	2	0	2	
Insurance status				
Insured	34	17	17	1
Uninsured	0	0	0	
Unknown	0	0	0	
Marital status				
Married	16	8	8	1
No married*	16	8	8	
Unknown	2	1	1	

AJCC, American Joint Committee on Cancer.

* indicates those divorced, single, or widowed.
